# Uncoupling of Longevity and Telomere Length in *C. elegans*

**DOI:** 10.1371/journal.pgen.0010030

**Published:** 2005-09-02

**Authors:** Marcela Raices, Hugo Maruyama, Andrew Dillin, Jan Karlseder

**Affiliations:** 1 The Salk Institute for Biological Studies, La Jolla, California, United States of America; 2 Graduate School of Biostudies, Kyoto University, Kyoto, Japan; Stanford University School of Medicine, United States of America

## Abstract

The nematode *Caenorhabditis elegans,* after completing its developmental stages and a brief reproductive period, spends the remainder of its adult life as an organism consisting exclusively of post-mitotic cells. Here we show that telomere length varies considerably in clonal populations of wild-type worms, and that these length differences are conserved over at least ten generations, suggesting a length regulation mechanism in *cis*. This observation is strengthened by the finding that the bulk telomere length in different worm strains varies considerably. Despite the close correlation of telomere length and clonal cellular senescence in mammalian cells, nematodes with long telomeres were neither long lived, nor did worm populations with comparably short telomeres exhibit a shorter life span. Conversely, long-lived *daf-2* and short-lived *daf-16* mutant animals can have either long or short telomeres. Telomere length of post-mitotic cells did not change during the aging process, and the response of animals to stress was found independent of telomere length. Collectively, our data indicate that telomere length and life span can be uncoupled in a post-mitotic setting, suggesting separate pathways for replication-dependent and -independent aging.

## Synopsis

The worm *Caenorhabditis elegans* has historically been used as a powerful model to study organismal aging. After a brief reproductive period, an adult worm consists of 959 post-mitotic cells. Telomeres, the natural ends of linear chromosomes, have long been implicated in the aging process of mitotic cells, and telomere shortening has been proposed to be a limiting factor in cell division. The authors show that telomere length of *C. elegans* chromosomes is independent of organismal aging. Worm telomeres were characterized in detail, and found to vary considerably in individual clones. These differences were conserved over several populations, suggesting that telomere length is controlled by similar mechanisms in worms and mammals, emphasizing the value of *C. elegans* as a model for telomere biology. However, telomere length has not been found to determine the potential life span of the animals, since worms with short telomeres lived as long as worms with long telomeres. Vice versa, worms that have an altered life span due to mutations in the insulin receptor pathway do not show any changes in telomere length. Telomere length was also found to be constant in isolated, aging worm populations, suggesting that organismal aging can be uncoupled from mitotic aging.

## Introduction

Telomeres, the specialized nucleoprotein structures that protect chromosome ends from degradation and fusions, have long been implicated in the replicative aging process of dividing cells [1–3]. Cellular aging in mitotic cultures is defined by initial telomere length, and the rate of telomere shortening per division, in the absence of telomerase. Human cells lose approximately 100 basepairs of telomeric repeats per cell division, and can undergo 50 to 80 doubling cycles until they enter a terminally differentiated stage, called replicative senescence.

In the mouse, it is well established that telomere maintenance and protection play a key role in the germline during early development and in highly proliferative organs [4,5]. However, telomere function in terminally differentiated cells, and the implications for post-mitotic senescence and organismal longevity remain unclear. The mouse model is not optimally suited to study the relation between organismal longevity and telomeres, considering that the cells of rapidly proliferating organs are strongly affected by telomere attrition in late generations by the targeted deletion of the telomerase RNA subunit, potentially masking the effects of short telomeres on differentiated cells. Nonetheless, it has been reported that the short telomeres in wild-derived strains of mice have no inverse effect on longevity, suggesting that organismal life span in this setting is independent of telomere length [6].

The nematode *Caenorhabditis elegans* does not suffer from the drawbacks of the mammalian systems, making it an optimal system to investigate a potential telomere-organismal life span relationship. Fully developed adult *C. elegans* hermaphrodites consist of 959 non-dividing somatic cells. To date, there is no example of cellular replication in adult *C. elegans*, although endoreduplication of nuclear DNA within a few hypodermal cells has been noted [7]. *C. elegans* therefore represents a unique model system to study the importance of telomere function in a post-mitotic setting and its implications on organismal aging.

The telomeric repeat sequence in the nematode *C. elegans* (TTAGGC) is similar to the telomeric sequence in mammals (TTAGGG), and this sequence has been found to be sufficient for the successful capping of chromosomes [8]. Few proteins that bind to nematode telomeres have been identified so far [9–12]. Worm telomere function has been analyzed only in the context of cell division, and, as for other higher eukaryotes, telomere maintenance has been shown to be essential for genome stability in the germline [13]. The relationship between organismal life span and telomere length in the worm has been controversial. *clk-2,* a gene with homology to *Saccharomyces cerevisiae* Tel2p, acts in the DNA damage response pathway and has been suggested to have little effect on telomere length [14]. In a similar study, *clk-2* mutations have been suggested to alter telomere length [15,16] while at the same time conferring a longer life span to the animals [17]. On the other hand, it has been proposed that the overexpression of the RNP A1 homolog *hrp-1* leads to telomere elongation, which in turn allows for a modest elongation in life span [12]. At this point, it is unclear whether the effects on life span are due to altered expression profiles or altered telomere length in the *hrp-1* overexpressing strains.

Here, we characterize *C. elegans* telomeres in detail in clones derived from individual worms, observe the life term of clonal populations and worm strains with different telomere length settings, investigate the effect of life span–altering mutations on telomere length, and describe the influence of telomere length on stress resistance.

## Results/Discussion

### Telomere Length in *C. elegans* Is Clonal and Inherited

**Figure 1:**
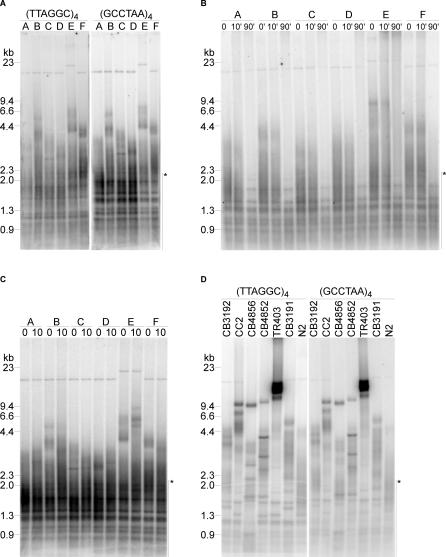
Clonal Variation and Conservation of Telomere Length in *C. elegans* (A) AluI/MboI digests of genomic DNA from six Bristol N2 clones was separated by gel electrophoresis and hybridized with (TTAGGC)_4_ or (GCCTAA)_4_ probes as described [28]. Fragment size is indicated in kb, and internal repeats are indicated by an asterisk. (B) DNA from six individual N2 clones was incubated with Bal31 for the indicated time, and then processed as in (A). (C) Six individual N2 clones were propagated for ten generations, and telomere length was determined as described. (D) Seven different *C. elegans* strains were examined for their telomere length as described in (A).

To study telomere function in *C. elegans,* we first determined the extent of telomere variation among wild-type worms. Telomere length was analyzed in clonal populations isolated from the Bristol N2 strain [18]. The length of terminal restriction fragments was determined by hybridizing (TTAGGC)_4_ and (GCCTAA)_4_ probes to *C. elegans* genomic DNA digested with AluI and MboI, restriction enzymes that liberate the chromosome ends. Telomere length ranged from 2 to 9 kilobasepairs (kb), and was heterogeneous among different clones (Figure 1A). While most isolated N2 clones contained fairly short telomeres in the size range of 2.5–3 kb (note clones A, C, D, and F), some clones showed bands of higher molecular weight, up to 9 kb (note clones B and E). Exonuclease Bal31 treatment prior to restriction fragmentation of genomic DNA samples confirmed that these bands represented terminal repeats and corresponded to telomeres (Figure 1B), as has been suggested earlier [8]. Within 10 min after treatment with Bal31, signal intensity was reduced, and the hybridization signal was almost completely removed after 90 min, indicating that the signal originated from DNA termini (Figure 1B), and pointing out the background signal resulting from internal cross-hybridizing sequences. These results are consistent with mammalian telomeres of similar length [19].

Given the telomere length differences between individual clonal worm populations, we next examined whether variations in telomere length were stably maintained throughout generations. Single worms were isolated to create individual, isolated colonies of worms. Because *C. elegans* exists almost exclusively as a self-fertile hermaphrodite, we grew each clonal population for several generations. Each of the clones was expanded and split into two. One part was immediately harvested and used for genomic DNA extraction for telomere length analysis; the other part was subsequently passaged for ten more population doublings, and the resulting worms were harvested for genomic DNA extraction. We found that following this regiment, even after ten generations the overall telomere length of each clonal population remained relatively constant, suggesting that individual telomere length regulation mechanisms are stably maintained and genetically conserved (Figure 1C).

The results of our last experiment suggested that telomere length among a population of worms was variable, yet stable. Consistent with this hypothesis, single telomere analysis of the left arm of Chromosome five (VL) between different, genetically isolated, wild-type *C. elegans* strains exhibited strong variations in telomere length, suggesting that length heterogeneity among strains could also exist at other chromosome ends [20]. We expanded this study to analyze the bulk telomere length in mixed populations of seven different wild-type worm strains, including the standard N2 worms. Telomere length distributions were different in all the strains examined (Figure 1D and Table 1). The average telomere length ranged from 2.6 kb in wild-type N2 worms to 16 kb in TR403 worms, with maximal telomere length reaching 7 kb in N2 worms and 17.8 kb in TR403 worms. Bal31 digests confirmed that the signal resulted from terminal repeats (unpublished data). These data suggest that both Southern blotting and single telomere analysis are appropriate methods for telomere length measurement in *C. elegans*. Altogether, these results demonstrate that there is strong heterogeneity in telomere length within individual *C. elegans* clones, as well as between different *C. elegans* strains.

**Table 1:**
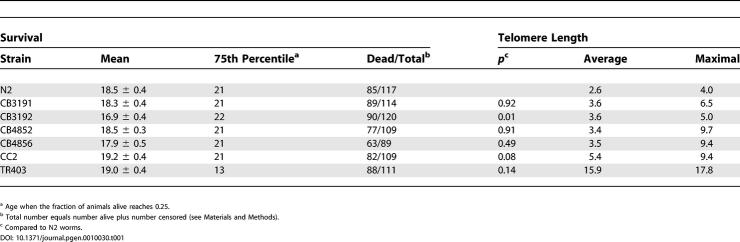
Survival, Average and Maximal Telomere Length of Different Worm Strains

### Post-Mitotic Life Span Is Independent of Telomere Length

Because it has been suggested that telomere length may play a role in organismal aging [5] and, more recently, post-mitotic aging of *C. elegans* [12], we tested whether the large variations in telomere length in the different wild-type worm strains analyzed were reflected in the life span of these animals. Despite the observed differences in telomere length, no significant variations in life span were detected among any of the seven worm strains (Table 1). Even TR403 worms with very long telomeres in the range of 16 kb displayed life spans that were not significantly different (*p* = 0.14) from those of standard Bristol N2 worms (Table 1), suggesting that long telomeres do not convey a noticeable survival advantage for the nematodes.

**Figure 2:**
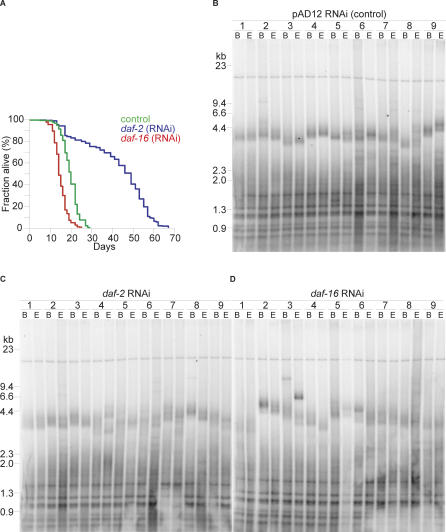
Organismal Life Span Is Independent of Telomere Length (A) Life span of N2 worms under control (green), *daf-2* (blue), and *daf-16* (red) RNAi suppression conditions. Worms were grown on bacteria expressing RNAi constructs targeting the indicated genes, and life span was assessed as described [21]. (B) Telomere length of nine individual control N2 clones was assessed as described. Samples were collected at the beginning (B) and end (E) of the experiment, and fragment size is indicated in kb. (C) Telomere length of nine individual *daf-2* (RNAi) N2 clones was assessed as described in (B). (D) Telomere length of nine individual *daf-16* (RNAi) N2 clones was assessed as described in (B).

To further establish if there was any correlation between life span regulation and telomere length, we analyzed whether variations in the expression of *daf-2* or *daf-16,* two key components of the insulin/IGF-1 signaling pathway known to be involved in *C. elegans* life span regulation [21,22], affected telomere length. To control for possible variation due to genetic drift that could accompany *daf-2* and *daf-16* mutant worms, we utilized a single wild-type strain, N2, and RNAi directed toward either *daf-2* or *daf-16* [23]. This approach ensured genetic identity among the worms examined in our experiment. Single worms were isolated to create individual colonies. Each clonal population was grown on the different bacteria expressing dsRNA of either *daf-2* or *daf-16* and divided into three parts. One part was immediately harvested and used for genomic DNA extraction, one part was used for life span analysis, and another part was subsequently passaged to analyze telomere length at the end of the life span. N2 worms grown on control bacteria displayed a mean life span of 19.8 + 0.5 d. However, in all clones analyzed, *daf-2* (RNAi) increased the mean life span to 42.3 + 2.1 d (*p* < 0.0001), and *daf-16* (RNAi) led to a significant decrease in life span (14.9 + 0.5 d, *p* < 0.0001) (Figure 2A and Table 2). Telomere length in nine individual clones was determined at the beginning (F1) and at the end of every experiment (Figure 2B–D). Since the life span of control *daf-16* (RNAi) and *daf-2* (RNAi) worms differs considerably, the end of the experiment correlates to F5 for control, F4 for *daf-16,* and F8 for *daf-2* animals. Despite the significant changes in survival, no correlation between life span and telomere length could be observed (Table 2). Average telomere length between control worms (2.5 kb), *daf-2* (RNAi) worms (2.5 kb), and *daf-16* (RNAi) worms (2.7 kb) did not vary, and the maximal telomere length settings did not correlate with long life span (Table 2). The longest clonal telomere length setting (> 6.5 kb), as well as the shortest maximal telomere length setting (3.5 kb) were both observed in short-lived *daf-16* (RNAi) treated worms (*daf-16* [RNAi] N2 clone 2 and clone 9, respectively).

**Table 2:**
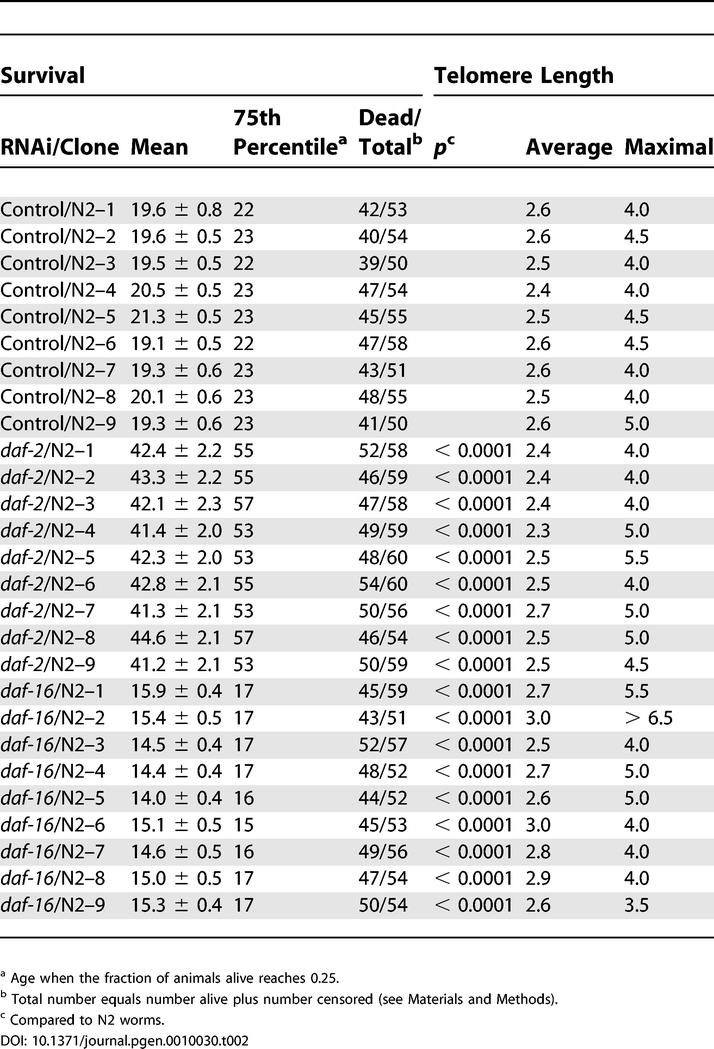
Survival, Average and Maximal Telomere Length of Control, *daf-2* (RNAi) and *daf-16* (RNAi) Clones

To verify whether the results gathered by suppression of *daf-2* and *daf-16* by RNAi could be confirmed in a mutant background, we analyzed nine individual clones from long-lived *daf-2(e1370)* and short-lived *daf-16(mu86)* null mutant animals for life span and telomere length. Wild-type N2 clones displayed an average life span of 18 + 0.9 d, all the different clones isolated from the *daf-2(e1370)* strain had an extended average life span of 37.7 + 1.7 d (*p* < 0.0001), and all the *daf-16(mu86)* null mutant clones lived significantly shorter than wild-type N2 worms, with an average life span of 12.9 + 0.5 d (*p* < 0.0001) (Figure S1A and Table S1). Telomere length analysis of each individual clone was performed, and the average and maximal telomere length settings were determined (Figure S1B–D). Despite the significant differences in life span, no obvious correlation between life span regulation and telomere length could be found (Table S1). Long-lived *daf-2(e1370)* mutant animals had an average telomere length of 3.8 kb, and short-lived *daf-16(mu86)* null mutant animals had an average telomere length of 3.7 kb. Control animals were observed to have slightly shorter telomeres than *daf-2(e1370)* and *daf-16(mu86)* mutant animals (Table S1). This is most likely traceable to the original clones used to generate the null mutants. Collectively, these data indicate two important aspects of aging and telomere length regulation. One, life span of nematodes can be manipulated independently of telomere length. Two, telomere length settings do not influence life span in the nematode.

### Telomere Length Remains Constant during Aging

**Figure 3:**
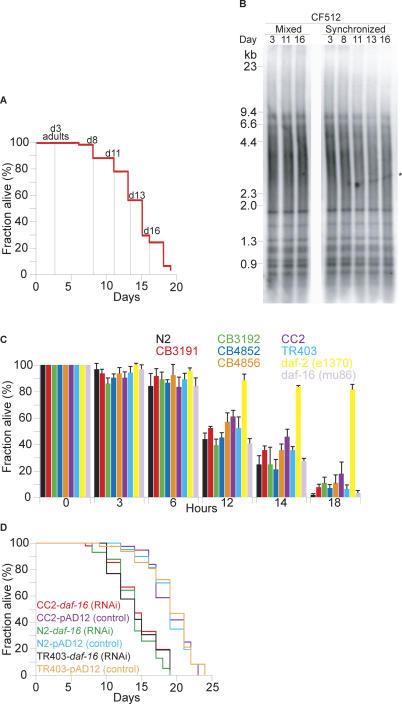
Telomere Length Is Independent from Organismal Life Span and Thermo Tolerance (A) CF512 temperature-sensitive sterile worms were grown at 25 °C, and life span was monitored. Samples were taken at indicated times. (B) DNA from CF512 temperature-sensitive sterile worms was extracted at the indicated time points, and telomere length was monitored in mixed and synchronous populations (for details, see Materials and Methods). Telomere length is indicated in kb. The asterisk points out a variable band-like signal resulting from gel drying. (C) Seven different worm strains and *daf-2* and *daf-16* RNAi suppressed worms (indicated in different colors) with genetically determined telomere length (Figure 1D) were subjected to heat treatment as described [31]. Survival was assessed at the indicated time points. (D) Two worm strains with long telomeres (CC2, TR403) and N2 worms with short telomeres were subjected to *daf-16* suppression by RNAi. Life spans were determined as described.

Next, we took advantage of CF512 *fer-15(b26);fem-1(hc17)* temperature-sensitive sterile worms [24] to test whether telomere length changes during organismal aging of an adult worm population. CF512 larvae were plated at the restrictive temperatures (25 °C) to block progeny production, and adult worms were harvested at 0, 5, 8, 10 and 13 d post reaching adulthood (Figure 3A). DNA was extracted from these samples, and telomere length was monitored by hybridization. In this model system that is free of contamination from embryos, no significant changes in telomere length were observed during the natural aging process of the animals (Figure 3B), again suggesting that organismal aging is independent of telomere shortening.

Worms usually exhibit an age-related failure to respond to heat shock, whereas long-lived worms are more resistant to thermal stress [25,26]. Furthermore, short telomere length in peripheral blood mononuclear cells has been correlated with high levels of stress in women [27]. We therefore tested whether there could be any associated stress resistance qualities that could be correlated with increased telomere length. Consequently, we studied thermal stress resistance in wild-type strains with varying telomere length settings, such as strain TR403, which has exceptionally long telomeres, as well as long-lived *daf-2(e1370)* mutant animals and short-lived *daf-16(mu86)* null mutant animals. No obvious correlation between thermo tolerance and telomere length could be found (Figure 3C). All the worm strains analyzed displayed similar survival rates at 35 °C, including the TR403 strain carrying 6-fold longer telomeres than the Bristol N2 laboratory strain. Only the *daf-2(e1370)* mutant worms, used as positive control, displayed significantly greater heat-shock resistance (Figure 3C, *p* < 0.0001); however, these animals do not have increased telomere length. In summary, these data indicate that long telomeres do not supply an advantage during times of thermal stress, and that thermo tolerance, supplied by longevity, does not correlate with telomere length. Two of these strains with long telomeres (CC2 and TR403), and the standard short telomere containing N2 worms were subsequently tested for their response to lower *daf-16* expression, and found to respond with a significantly shorter life span, independent of their telomere length, again suggesting that genetically controlled telomere length is uncoupled from genetically controlled organismal life span (Figure 3D).

Collectively our data argue for an uncoupling of organismal aging and telomere length. We have shown that both telomere length as well as longevity seem genetically controlled, but at this point our experiments do not exclude the possibility of epigenetic differences involved in the control of telomere length. However, both long-lived and short-lived worms can have either short or long telomeres. On the other hand, long telomeres do not contribute to increased longevity or stress resistance, as demonstrated in the TR403 or CC2 strains.

Telomerase knockout mice have progressively short telomeres after generational passaging. However, age-related effects in these mice are difficult to assess due to the increased rate of telomere loss in highly proliferative cells, compared to terminally differentiated cells. Our results suggest that telomere length does not influence the aging process in terminally differentiated, post-mitotic cells of an entire organism, *C. elegans*. Therefore, while telomere length control is essential for maintenance of the germline and other highly proliferative cells, telomere length control is dispensible for aging of non-dividing cells. We believe that other genetic pathways, such as the insulin/IGF-1 pathway, are required for the proper aging of post-mitotic cells.

Recently, it has been reported that elongation of telomeres by overexpression of *hrp-1,* a putative RNA binding protein that has been suggested to also associate with single stranded telomeric DNA, increased life span in a *daf-16* dependent manner. Furthermore, overexpression of *hrp-1* resulted in worms with longer telomeres and increased resistance to heat stress [12]. Here we show that telomere length can be uncoupled from organismal life span, is not dependent upon the insulin/IGF-1 signaling pathway, and is independent of resistance to thermal stress, suggesting that the effects that *hrp-1* overexpression has on nematode longevity are not due to telomere elongation. Therefore, *hrp-1* may be bifunctional, having one role in organismal aging and a separate, independent role in telomere length regulation.

By now, it is well established that telomere length and telomere shortening rates during mitotic cell division limit the replicative life span of mammalian cells. However, fibroblasts that enter a terminally differentiated state due to critically short telomeres usually do not die, but remain in a metabolically active state. In light of the experiments presented here, we suggest a model where telomere length and life span in post-mitotic organisms and cells are independent of each other.

## Materials and Methods

### Strains.

Worms were grown at 20 °C and maintained as described [18]. The strains used in this study include the following: Wild-type strains N2, CB3191, CB3192, CB4852, CB4856, CC2, TR403 (obtained from the Caenorhabditis Genetics Center, Minneapolis, Minnesota, United States) and the *daf-16(mu86)I* and *daf-2(e1370)III* mutant strains [22], as well as the temperature-sensitive sterile CF512 *fer-15(b26)II; fem-1(hc17)IV* strain [24].

### Telomere length analysis.

For genomic DNA extraction, nematodes were floated off the bacterial lawns with M9 buffer, centrifuged, and washed again twice with M9 buffer. After harvesting, worms were lysed for 1 h at 50 °C in buffer containing 100 mM Tris HCl (pH 8.5), 100 mM NaCl, 50 mM EDTA, 1% SDS, 100 μg/ml proteinase K, and 1% β-mercaptoethanol. DNA was isolated by phenol-chloroform extraction in Phase Lock Gel tubes (Eppendorf, Hamburg, Germany) and isopropanol precipitation. DNA was then treated with 10 μg/ml RNAse A for 2 h. After that, the proteinase K incubation and extraction were repeated once. DNA was dissolved in 10 mM Tris HCl (pH 7.5) and 0.1 mM EDTA and stored at 4 °C. Telomere blots were carried out as described [28]. (TTAGGC)_4_ and (GCCTAA)_4_ end-labeled oligonucleotides were used as probes. Blots were exposed to PhosphorImager screens, and mean telomeric restriction fragment lengths were determined using ImageQuant software (both from GE Healthcare, Little Chalfont, United Kingdom).

### Bal 31 treatments.

*C. elegans* DNA (50–100μg) was treated with 10–20 U of Bal 31 exonuclease (New England Biolabs, Beverly, Massachusetts, United States) at 30 °C in 500 μl of 1× Bal31 buffer. Reactions were stopped with the addition of EGTA (25 mM final concentration) at 0, 0.5, and 2 h. Samples were phenol extracted, and the DNA was collected by isopropanol precipitation. DNA from each time point was digested with AluI and MboI, quantified using Hoechst fluorimetry, fractionated on 0.7% agarose gels, and blotted as described above.

### RNAi.

HT115 bacteria expressing dsRNA were grown at 37 °C in LB with 10 μg/ml tetracycline and 50 μg/ml carbenicillin, then were seeded onto NG-carbenicillin plates and supplemented with 100 μl 0.1 M IPTG (1.5 mM final concentration). Eggs were added to plates, and animals were transferred to new plates every 3 d. Vectors used in this study were: pAD12 (control vector Bluescript with opposing T7 promoters), pAD48 (2.3 kb of *daf-2* coding sequence cloned into pAD12), and pAD43 (1.1 kb of *daf-16* coding sequence cloned into pAD12).

### Survival analysis.

Life span assays were performed at 20 °C and were initiated at the L1 larvae stage [21]. Animals (60–120 per experiment) were transferred away from their progeny to new plates every other day until the end of the reproductive period. We used Statview 4.5 (SAS, Cary, North Carolina, United States) software to carry out statistical analysis and to determine mean life spans and 75th percentiles. The logrank (Mantel-Cox) statistics were used to evaluate the hypothesis that animals in experimental and control groups behaved similarly. Animals that crawled off the plate, “exploded” (i.e., had a gonad extruding through their vulva), or “bagged” (i.e., died from internal hatching) were censored at the time of the event and were incorporated into the dataset as described [29].

### Survival analysis of temperature-sensitive sterile animals.

Age-synchronized worms were obtained at 25 °C using the CF512 *fer-15(b26)II;fem-1(hc17)IV* temperature-sensitive sterile strain [24]. Embryos were isolated using an alkaline hypochlorite solution [30] from CF512 worms grown at 15 °C. Synchronized L1 larvae were isolated by gently shaking the embryos overnight at room temperature in M9 buffer. L1 larvae were placed on fresh OP50 plates and incubated at 25 °C until the end of the experiment. Adult worms were harvested at different time points, and telomere length was analyzed as described. At each time point, all worms were transferred to fresh OP50 plates. In parallel, a small fraction of the synchronized L1 larvae was placed on fresh OP50 plates and incubated at 20 °C to produce a mixed population of worms that was maintained throughout the experiment and used as control. Survival analyses of CF512 were performed at 25 °C as described.

### Thermo tolerance assays.

All the worms were maintained under standard *C. elegans* growth condition at 20 °C. Response to heat shock was assayed on age-synchronized groups, produced by placing reproductive adults onto fresh agar plates and permitting the eggs laid to develop for 4 d into adults [31]. Survival time at elevated temperatures was determined by shifting synchronized d 4 adults (10–15 per plate) to 35 °C. The first time point was taken approximately 6 h after the shift, with subsequent time points taken every 3 h.

## Supporting Information

Figure S1 Organismal Life Span Is Independent of Telomere Length (A) Life span of control N2 worms (green), *daf-2(e1370)* mutants (blue), and *daf-16(mu86)* mutants (red). Life span was assessed as described [21].
(B) Telomere length of nine individual control N2 clones was assessed as described. Samples were collected at the beginning (B) and end (E) of the experiment, and fragment size is indicated in kb.
(C) Telomere length of nine individual *daf-2(e1370)* mutant clones was assessed as described in (B).
(D) Telomere length of nine individual *daf-16(mu86)* mutant clones was assessed as described in (B).
(6.4 MB TIF)

Table S1 Survival, Average and Maximal Telomere Length of Control *daf-2(e1370)* Mutants and *daf-16(mu86)* Mutant Clones (52 KB DOC)

## Abbreviation

kb: kilobasepair
