# Correction: Uncoupling of Longevity and Telomere Length in *C. elegans*

**DOI:** 10.1371/journal.pgen.0010081

**Published:** 2005-12-30

**Authors:** Marcela Raices, Hugo Maruyama, Andrew Dillin, Jan Karlseder

In *PLoS Genetics,* volume 1, issue 3, DOI: 10.1371/journal.pgen.0010030

Andrew Dillin should be listed as a corresponding author (in addition to Jan Karlseder). E-mail: dillin@salk.edu

