# Supplementary material for: Uncoupling of Longevity and Telomere Length in C. elegans
Source: PLoS Genet. 2005 Sep 2;1(3):e30. doi: 10.1371/journal.pgen.0010030 (PMC1200426; doi:10.1371/journal.pgen.0010030)
Supplement: Supplementary file 1 [file pgen.0010030.st001.doc]

Raices et al.,

Suppl. Table 1

Table S1	Survival and telomere length of *daf-2* and *daf-16* mutant strains

Survival	Telomere length

| Strain | Mean | 75^th^%a | Dead/total | Pc | average | maximal |
| --- | --- | --- | --- | --- | --- | --- |
| N2-1 | 19.4+/-0.8 | 23 | 45/58 |  | 2.3 | 5.0 |
| N2-2 | 18.9+/-0.8 | 22 | 47/60 |  | 2.3 | 5.0 |
| N2-3 | 17.6+/-0.9 | 22 | 41/60 |  | 2.5 | 4.0 |
| N2-4 | 19.3+/-1.2 | 25 | 45/61 |  | 2.3 | 4.0 |
| N2-5 | 17.3+/-0.8 | 22 | 51/61 |  | 2.4 | 3.5 |
| N2-6 | 17.3+/-0.8 | 21 | 47/53 |  | 2.5 | 6.0 |
| N2-7 | 17.6+/-1.0 | 23 | 49/60 |  | 2.3 | 4.0 |
| N2-8 | 17.0+/-1.1 | 21 | 43/61 |  | 2.4 | 5.0 |
| N2-9 | 17.3+/-0.8 | 21 | 48/60 |  | 2.3 | 4.0 |
| *daf-2(e1370)-*1 | 42.0+/-1.6 | 53 | 48/60 | <0.0001 | 3.9 | >6.5 |
| *daf-2(e1370)-*2 | 36.2+/-1.6 | 42 | 44/59 | <0.0001 | 3.7 | 5.0 |
| *daf-2(e1370)-*3 | 37.1+/-1.8 | 46 | 46/61 | <0.0001 | 3.5 | 4.0 |
| *daf-2(e1370)-*4 | 36.1+/-1.7 | 42 | 48/65 | <0.0001 | 3.6 | 5.0 |
| *daf-2(e1370)-*5 | 41.6+/-1.4 | 50 | 45/60 | <0.0001 | 4.0 | 5.0 |
| *daf-2(e1370)-*6 | 34.4+/-2.0 | 46 | 42/60 | <0.0001 | 3.7 | 4.0 |
| *daf-2(e1370)-*7 | 37.0+/-1.8 | 48 | 46/62 | <0.0001 | 3.7 | 5.0 |
| *daf-2(e1370)-*8 | 37.5+/-1.7 | 48 | 52/62 | <0.0001 | 4.1 | 4.0 |
| *daf-2(e1370)-*9 | 37.7+/-1.6 | 46 | 48/61 | <0.0001 | 4.2 | 4.0 |
| *daf-16(mu86)*-1 | 13.5+/-0.5 | 16 | 48/59 | <0.0001 | 4.7 | >6.5 |
| *daf-16(mu86)*-2 | 13.6+/-0.3 | 16 | 43/51 | <0.0001 | 4.0 | >6.5 |
| *daf-16(mu86)*-3 | 12.2+/-0.4 | 13 | 48/50 | <0.0001 | 2.9 | 6.5 |
| *daf-16(mu86)*-4 | 12.3+/-0.4 | 13 | 42/52 | <0.0001 | 4.5 | >6.5 |
| *daf-16(mu86)*-5 | 12.0+/-0.4 | 14 | 43/53 | <0.0001 | 2.7 | 3.5 |
| *daf-16(mu86)*-6 | 12.9+/-0.3 | 14 | 41/58 | <0.0001 | 2.7 | 3.5 |
| *daf-16(mu86)*-7 | 13.4+/-0.4 | 14 | 43/57 | <0.0001 | 2.8 | >6.5 |
| *daf-16(mu86)*-8 | 13.2+/-0.5 | 16 | 47/60 | <0.0001 | 4.3 | >6.5 |
| *daf-16(mu86)*-9 | 13.2+/-0.5 | 16 | 46/59 | <0.0001 | 3.8 | >6.5 |

^a^Age when the fraction of animals alive reaches 0.25

^b^Total number equals number alive plus number censored (see methods section)

^c^Compared to N2 worms
